# Production of Protocatechuic Acid from *p*-Hydroxyphenyl (H) Units and Related Aromatic Compounds Using an Aspergillus niger Cell Factory

**DOI:** 10.1128/mBio.00391-21

**Published:** 2021-06-22

**Authors:** Ronnie J. M. Lubbers, Ronald P. de Vries

**Affiliations:** a Fungal Physiology, Westerdijk Fungal Biodiversity Institute, Utrecht University, Utrecht, The Netherlands; b Fungal Molecular Physiology, Utrecht University, Utrecht, The Netherlands; Universidade de Sao Paulo

**Keywords:** 3,4-dihydroxybenzoic acid, benzoic acid metabolic pathway, caffeic acid, fungi, *p*-coumaric acid, protocatechuate hydroxylase, *Aspergillus niger*, filamentous fungi

## Abstract

Protocatechuic acid (3,4-dihydroxybenzoic acid) is a chemical building block for polymers and plastics. In addition, protocatechuic acid has many properties of great pharmaceutical interest. Much research has been performed in creating bacterial protocatechuic acid production strains, but no protocatechuic acid-producing fungal cell factories have been described. The filamentous fungus Aspergillus niger can produce protocatechuic acid as an intermediate of the benzoic acid metabolic pathway. Recently, the *p*-hydroxybenzoate-*m*-hydroxylase (*phhA*) and protocatechuate 3,4-dioxygenase (*prcA*) of A. niger have been identified. It has been shown that the *prcA* deletion mutant is still able to grow on protocatechuic acid. This led to the identification of an alternative pathway that converts protocatechuic acid to hydroxyquinol (1,3,4-trihydroxybenzene). However, the gene involved in the hydroxylation of protocatechuic acid to hydroxyquinol remained unidentified. Here, we describe the identification of protocatechuate hydroxylase (decarboxylating) (PhyA) by using whole-genome transcriptome data. The identification of *phyA* enabled the creation of a fungal cell factory that is able to accumulate protocatechuic acid from benzyl alcohol, benzaldehyde, benzoic acid, caffeic acid, cinnamic acid, cinnamyl alcohol, *m-*hydroxybenzoic acid, *p*-hydroxybenzyl alcohol, *p-*hydroxybenzaldehyde, *p*-hydroxybenzoic acid, *p*-anisyl alcohol, *p*-anisaldehyde, *p*-anisic acid, *p*-coumaric acid, and protocatechuic aldehyde.

## INTRODUCTION

For centuries, oil and natural gas have been used to create fuels, plastics, and other raw materials. These fossil resources are becoming limited, while the demand for chemicals, fuels, and materials is still increasing. This has led researchers to look for alternative, preferably sustainable and renewable resources and to develop new methods to produce these chemicals, fuels, and materials. A promising technology is biorefining that uses microbial fermentations to produce chemical building blocks, fuels, and bioplastics (1–4). Currently, most aromatic compounds are produced using petroleum-based resources, but several aromatic compounds have already been produced through biorefining (2, 3). The aromatic compound protocatechuic acid (3,4-dihydroxybenzoic acid) is a chemical building block for polymers and plastics (5–7). In addition, protocatechuic acid has many pharmaceutical properties, such as antibacterial, anticancer, antidiabetic, antiulcer, antiviral, nematicidal, antiatherosclerotic, and antihyperlipidemic activities (8–14). The huge potential of this aromatic compound results in many new strategies for producing protocatechuic acid through bacterial and yeast cell factories (4, 15–18). However, no protocatechuic acid-producing filamentous fungal factories have been described so far, despite their large potential for aromatic conversion (19).

Protocatechuic acid can be metabolized through the benzoic acid metabolic pathway (19). In Aspergillus niger, benzoic acid is converted in three steps (20, 21). First, benzoic acid is *p*-hydroxylated to *p*-hydroxybenzoic acid by benzoate-4-monooxygenase (BphA) (22, 23). Then, *p*-hydroxybenzoic acid is *m*-hydroxylated to protocatechuic acid by *p*-hydroxybenzoate-*m*-hydroxylase (PhhA) (20, 21, 24). Finally, the aromatic ring of protocatechuic acid is cleaved to form 3-carboxy-*cis*,*cis*-muconic acid, which is catalyzed by protocatechuate 3,4-dioxygenase (PrcA). This compound is further degraded through the oxoadipate pathway (25). Recently, PhhA and PrcA of A. niger were identified (24). In the same study, deletion of the *prcA* gene resulted in a transient reduced growth of the strain on protocatechuic acid, revealing an alternative protocatechuic acid pathway. In order to prove the alternative pathway, a double deletion mutant of *prcA* and the hydroxyquinol 1,2-dioxygenase gene (*hqdA*) was created. Growth of this mutant on protocatechuic acid resulted in the accumulation of hydroxy-1,4-benzoquinone. Hydroxy-1,4-benzoquinone is the oxidative product of hydroxyquinol, indicating that protocatechuic acid is decarboxylated to hydroxyquinol by an unidentified protocatechuate hydroxylase. Recently, a salicylate hydroxylase (ShyA) converting 2-hydroxybenzoic acid (salicylic acid) to catechol was identified and characterized (26). ShyA was also able to convert 2,3-dihydroxybenzoic acid and 2,5-dihydroxybenzoic acid, but it did not convert protocatechuic acid to hydroxyquinol.

In this study, we identified the protocatechuate hydroxylase responsible for the formation of hydroxyquinol using whole-genome transcriptome data of A. niger grown on *p*-hydroxybenzoic acid, protocatechuic acid, *p-*coumaric acid, and caffeic acid. The Δ*phyA* Δ*prcA* double deletion mutant resulted in the accumulation of protocatechuic acid when grown on multiple benzoates and cinnamic acids.

## RESULTS

### Identification of candidate genes encoding putative PhyA.

To identify the gene encoding PhyA, transcriptome data of A. niger N402 grown on *p*-hydroxybenzoic acid, protocatechuic acid, caffeic acid, and *p*-coumaric acid were used (24). In the A. niger NRRL3 genome, 32 genes are annotated as salicylate 1-monooxygenase (salicylate hydroxylase). Of these genes, only the salicylate hydroxylase (ShyA, NRRL3_9273) gene and the AzaH (NRRL3_145) gene of the azaphilone metabolic gene cluster have been characterized (26, 27). Transcriptional data of the 32 salicylate 1-monooxygenases revealed that seven genes, including *shyA*, were induced (fragments per kilobase per million [FPKM],  ≥10; fold change, ≥2; *P* value, ≤0.01) by at least one of the tested aromatic compounds (Table 1). However, only one gene (NRRL3_4659) was induced by all four aromatic compounds and was also the most highly induced salicylate hydroxylase gene in the presence of protocatechuic acid and caffeic acid. Therefore, NRRL3_4659 was selected as the putative *phyA*.

**TABLE 1:** Transcriptome data of putative salicylate hydroxylases from A. niger induced by *p*-hydroxybenzoic acid, protocatechuic acid, *p*-coumaric acid, or caffeic acid^*a*^

Gene identifier	*p*-Hydroxybenzoic acid			Protocatechuic acid			*p*-Coumaric acid			Caffeic acid			No carbon source (FPKM)
	FPKM	FC	*P* value	FPKM	FC	*P* value	FPKM	FC	*P* value	FPKM	FC	*P* value	
NRRL3_04659	**42.6**	**2.74**	**0.00**	**484.2**	**6.16**	**0.00**	**77.0**	**3.75**	**0.00**	**752.9**	**7.01**	**0.00**	6.6
NRRL3_06695	16.4	0.88	0.04	**105.1**	**3.45**	**0.00**	27.8	1.78	0.00	21.5	1.43	0.00	9.5
NRRL3_09897	7.0	0.14	0.83	**33.6**	**2.25**	**0.00**	10.1	0.78	0.06	**32.5**	**2.43**	**0.00**	6.9
NRRL3_09295	**58.5**	**2.32**	**0.00**	42.6	1.81	0.00	**64.2**	**2.61**	**0.00**	**54.2**	**2.38**	**0.00**	12.6
NRRL3_08287	**227.0**	**4.30**	**0.00**	28.1	1.28	0.00	**116.3**	**3.51**	**0.00**	26.7	1.43	0.00	12.1
NRRL3_00043	3418.6	1.27	0.15	3080.5	1.06	0.26	**6491.6**	**2.19**	**0.00**	2136.2	0.80	0.35	1384.4
NRRL3_09723	20.4	0.84	0.01	20.3	0.78	0.03	**48.3**	**2.22**	**0.00**	27.4	1.43	0.00	12.2

a Values for genes induced (fragments per kilobase per million [FPKM] ≥ 10, fold change [FC] ≥ 2, *P* value ≤ 0.01) under these conditions are highlighted in bold. Fold change (log_2_ values) and *P* values were calculated using DeSeq2 (65).

### Double deletion of *phyA* and *prcA* results in reduced growth on protocatechuic acid.

To study the putative *phyA* in A. niger, single deletion (Δ*phyA*) and double deletion (Δ*phyA* Δ*prcA*) mutants were made. In addition, a Δ*prcA* mutant with a functional *pyrG* gene was made to obtain strains that are more similar to each other. The A. niger reference strain and Δ*phyA*, Δ*prcA*, Δ*hqdA*, Δ*phyA* Δ*prcA*, and Δ*prcA* Δ*hqdA* deletion strains were grown on multiple aromatic compounds as the sole carbon source.

**FIG 1:**
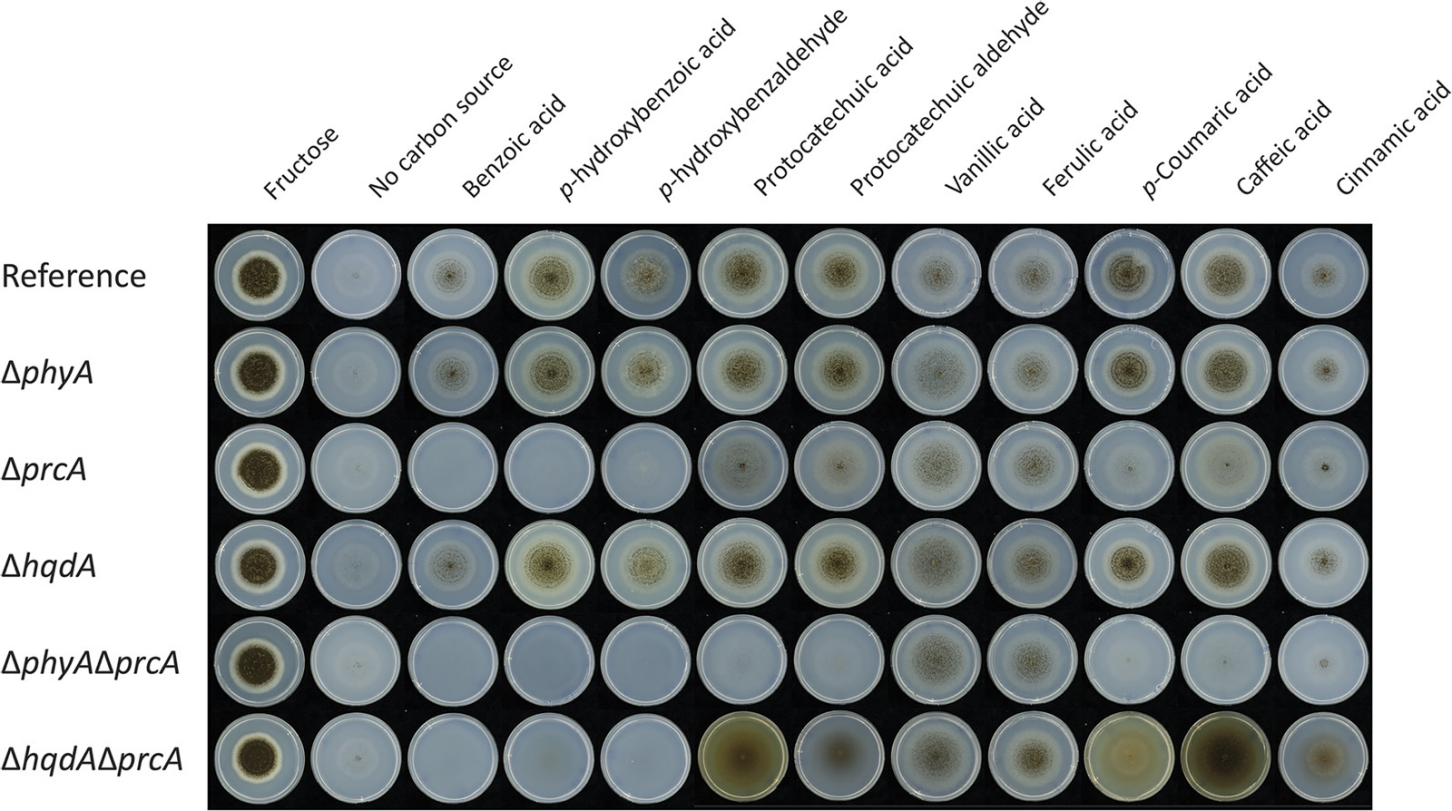
Growth profile of the A. niger reference strain and Δ*phyA*, Δ*prcA*, Δ*hqdA*, Δ*phyA* Δ*prcA*, and Δ*prcA* Δ*hqdA* deletion mutants on aromatic compounds. The plates were incubated at 30°C for 7 days.

No altered phenotypes were observed when Δ*phyA* or Δ*hqdA* strains were grown on the tested aromatic compounds in comparison to the reference strain (Fig. 1). An altered phenotype was observed when *prcA* was deleted together with *hqdA*, as reported previously (24). This is also the case for the combined deletion of *phyA* and *prcA*, and this strain has reduced growth compared to the Δ*prcA* strain. In addition, accumulation of hydroxy-1,4-benzoquinone was observed in the Δ*prcA* Δ*hqdA* strain on protocatechuic acid, but not in the Δ*phyA* Δ*prcA* strain, indicating that the hydroxylation of protocatechuic acid to hydroxyquinol is blocked. Accumulation of hydroxy-1,4-benzoquinone was also observed on cinnamic acid, caffeic acid, *p*-coumaric acid, and protocatechuic aldehyde by the Δ*prcA* Δ*hqdA* deletion strain, indicating that these compounds are converted to protocatechuic acid (Fig. 1).

### Accumulation of protocatechuic acid by A. niger Δ*phyA* Δ*prcA*.

An accumulation test was performed to verify whether the Δ*phyA* Δ*prcA* mutant can be used as a cell factory that accumulates protocatechuic acid from benzoic and cinnamic acids. Ferulic acid, vanillin, and veratric acid were included as negative controls. In addition, bitter almond oil, which consists mainly of benzaldehyde (28–31), and a mixture of five aromatic compounds consisting of benzaldehyde, benzoic acid, *p-*hydroxybenzoic acid, *p-*coumaric acid, and caffeic acid were tested.

After 24 h, all initial substrates were no longer detected in the culture medium of the strains. Accumulation of protocatechuic acid was observed in the culture medium of the Δ*phyA* Δ*prcA* mutant grown in medium with protocatechuic aldehyde, benzoic acid, benzaldehyde, benzyl alcohol, *p*-anisic acid, *p*-anisaldehyde, *p-*hydroxybenzoic acid, *p*-hydroxybenzaldehyde, *p*-hydroxybenzyl alcohol, *m-*hydroxybenzoic acid, *p-*coumaric acid, caffeic acid, cinnamic acid, and cinnamyl alcohol (Table 2). The Δ*phyA* Δ*prcA* strain grown in medium with the aromatic compound mixture also resulted in the accumulation of protocatechuic acid. No protocatechuic acid or other aromatic compound was observed when the strain was grown on anethole, ferulic acid, *p-*cresol, vanillin, or veratric acid. No accumulated compounds were detected in the supernatant of the culture medium of the reference strain, indicating that all aromatic compounds were utilized by this strain.

**TABLE 2:** Accumulation of protocatechuic acid by the Δ*phyA* Δ*prcA* mutant^*a*^

Substrate	Compound detected in reference strain	Δ*phyA* Δ*prcA* mutant	
		Concn (mM) of compound detected	Conversion rate (%)
Protocatechuic acid	–	1.68 ± 0.03	84.0
Protocatechuic aldehyde	–	1.95 ± 0.11	97.5
Benzoic acid	–	1.60 ± 0.02	80.0
Benzaldehyde	–	1.95 ± 0.10	97.5
Benzyl alcohol	–	1.78 ± 0.15	88.9
*p*-Anisic acid	–	1.66 ± 0.11	83.0
*p*-Anisaldehyde	–	2.09 ± 0.02	≥99.0
*p*-Anisyl alcohol	–	1.63 ± 0.11	81.5
*p*-Hydroxybenzoic acid	–	1.81 ± 0.05	90.5
*p*-Hydroxybenzaldehyde	–	2.10 ± 0.06	≥99.0
*p*-Hydroxybenzyl alcohol	–	1.90 ± 0.02	95.0
*m*-Hydroxybenzoic acid	–	1.69 ± 0.20	84.5
*p*-Coumaric acid	–	1.82 ± 0.05	91.0
Caffeic acid	–	2.10 ± 0.09	≥99.0
Cinnamyl alcohol	–	0.83 ± 0.03	41.5
Cinnamic acid	–	0.28 ± 0.03	13.7
Ferulic acid	–	–	–
Vanillin	–	–	–
Veratric acid	–	–	–
*p-*Cresol	–	–	–
Anethole	–	–	–
Bitter almond oil^*b*^	–	1.63 ± 0.04	81.5
Mixture^*c*^	–	10.32 ± 0.31	≥99.0

a The protocatechuic acid concentrations were determined from the average of biological triplicates. A 2 mM concentration was used as the starting substrate. –, no compound detected.

b Bitter almond oil consists mainly of benzaldehyde (96% to 98%) (28–31); the molecular weight of benzaldehyde was used to obtain approximately 2 mM.

c The mixture consisted of 2 mM benzaldehyde, 2 mM benzoic acid, 2 mM *p-*hydroxybenzoic acid, 2 mM *p-*coumaric acid, and 2 mM caffeic acid.

### The Δ*phyA* Δ*prcA* mutant undergoes cell lysis when grown in medium with *p*-coumaric acid, *p*-hydroxybenzoic acid, and protocatechuic acid but not in medium with ferulic acid.

**FIG 2:**
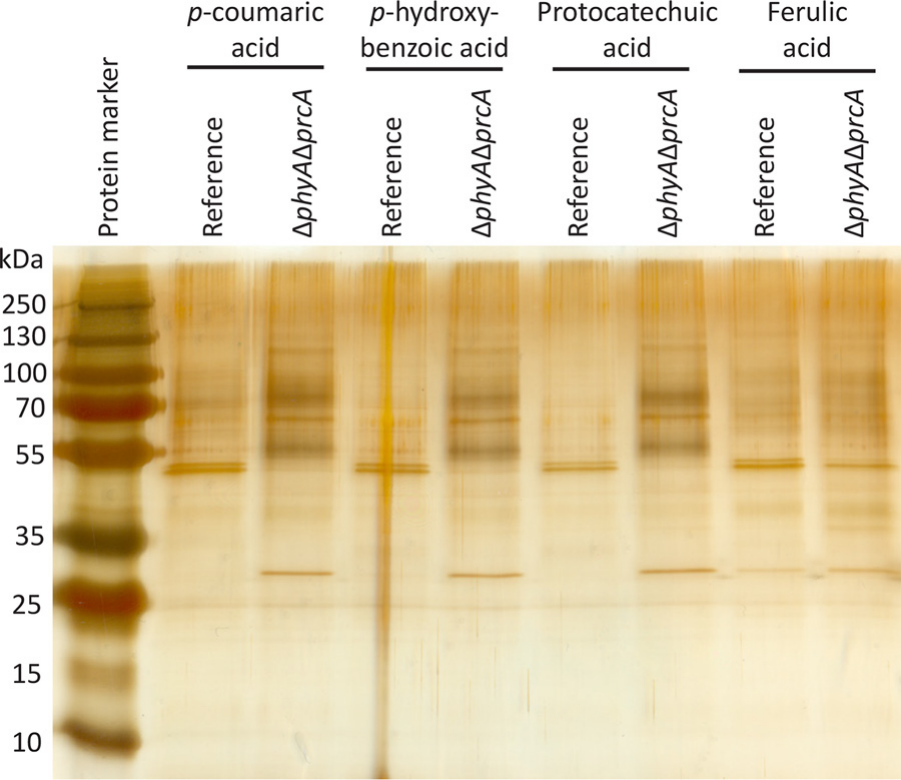
SDS-PAGE profile of the Δ*phyA* Δ*prcA* mutant and the reference strain after growth in MM with *p-*coumaric acid, *p-*hydroxybenzoic acid, protocatechuic acid, and ferulic acid. Equal amounts of supernatant were loaded on the SDS-PAGE gel and stained by silver staining.

**FIG 3:**
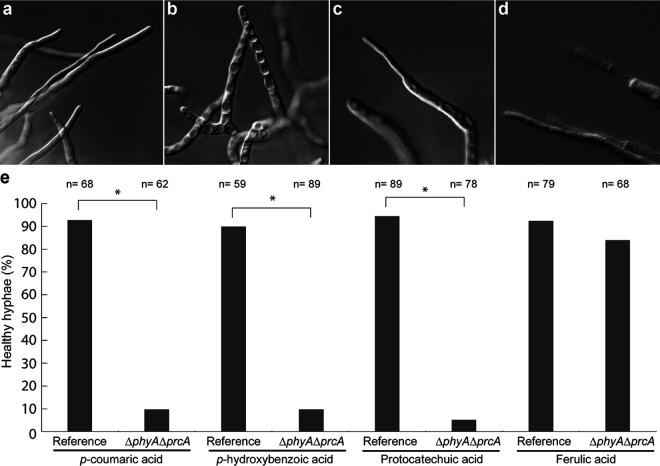
Microscopical observations of the Δ*phyA* Δ*prcA* mutant and the reference strain grown in MM with *p-*coumaric acid, *p-*hydroxybenzoic acid, protocatechuic acid, and ferulic acid. Examples are shown of healthy hyphae (a), hyphae with increased numbers of vacuoles (b), thinning of hyphae (c), and plugging after hyphae wounding (d). (e) Healthy and stressed hyphae from the reference and mutant strains were counted. Significant differences were determined by the chi-square test (α = 0.01) and are marked with an asterisk.

To determine if the release of protocatechuic acid by the Δ*phyA* Δ*prcA* mutant is caused by cell lysis due to starvation, supernatant samples from the accumulation experiments were analyzed by SDS-PAGE. The supernatant samples of the reference and Δ*phyA* Δ*prcA* strains grown in *p-*coumaric acid, protocatechuic acid, *p*-hydroxybenzoic acid, and ferulic acid were loaded on an SDS-PAGE gel and visualized by silver staining (Fig. 2). All supernatant samples of the reference strain are similar. Only the supernatant sample of the Δ*phyA* Δ*prcA* strain grown on ferulic acid is similar to the reference samples, while under the other conditions they are not similar and appear to have more proteins in the supernatant. In addition, changes in the appearance of hyphae were observed (Fig. 3). The hyphae from the Δ*phyA* Δ*prcA* strain grown in *p-*coumaric acid, protocatechuic acid, and *p*-hydroxybenzoic acid were stressed or lysing (Fig. 3). When grown in ferulic acid, the hyphae from the Δ*phyA* Δ*prcA* strain were similar to those of the reference (Fig. 3e). Next to that, the pH of the medium of the Δ*phyA* Δ*prcA* strain was significantly higher than that of the reference (see Table S4 in the supplemental material). These results suggest that cell lysis occurred in the Δ*phyA* Δ*prcA* strain, likely caused by starvation, which resulted in the release of intracellular fluid.

10.1128/mBio.00391-21.5 TABLE S4 pH measurement of the supernatant samples from the reference and Δ*phyA* Δ*prcA* strains. Average and standard deviation values were calculated from biological triplicates. Download Table S4, XLSX file, 0.01 MB. Copyright © 2021 Lubbers and de Vries. 2021 Lubbers and de Vries. https://creativecommons.org/licenses/by/4.0/ This content is distributed under the terms of the Creative Commons Attribution 4.0 International license.

### PhyA converts protocatechuic acid.

**FIG 4:**
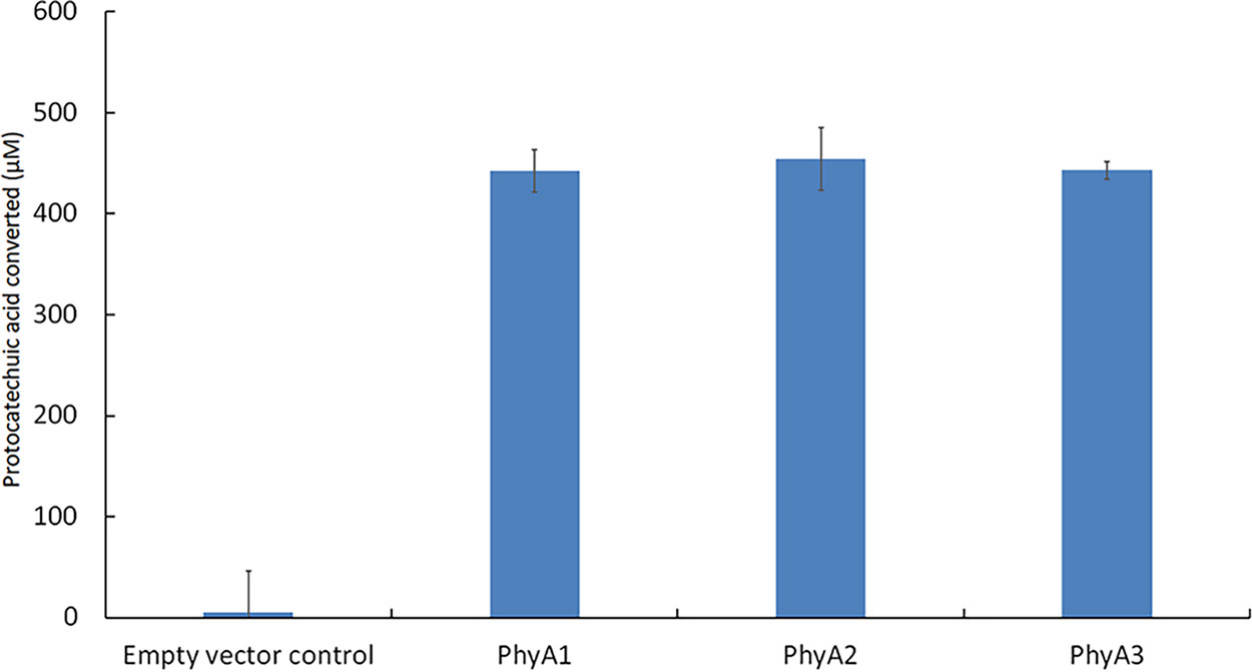
Amount of protocatechuic acid converted by PhyA-containing cell extracts. The reaction mixtures contained 500 μM protocatechuic acid and were incubated for 1 h at 30°C.

To confirm the enzymatic activity of PhyA, an Escherichia coli strain that produces PhyA was created. Cell-free extracts of three independent recombinant strains (PhyA.1, PhyA.2, and PhyA.3) and a strain harboring the pET23 plasmid without insert were collected and tested for activity on protocatechuic acid. After overnight incubation, all reaction mixtures were analyzed by high-performance liquid chromatography (HPLC) (Fig. 4). The protocatechuic acid concentration of the reaction mixtures containing PhyA were significantly reduced compared to the empty vector control.

**FIG 5:**
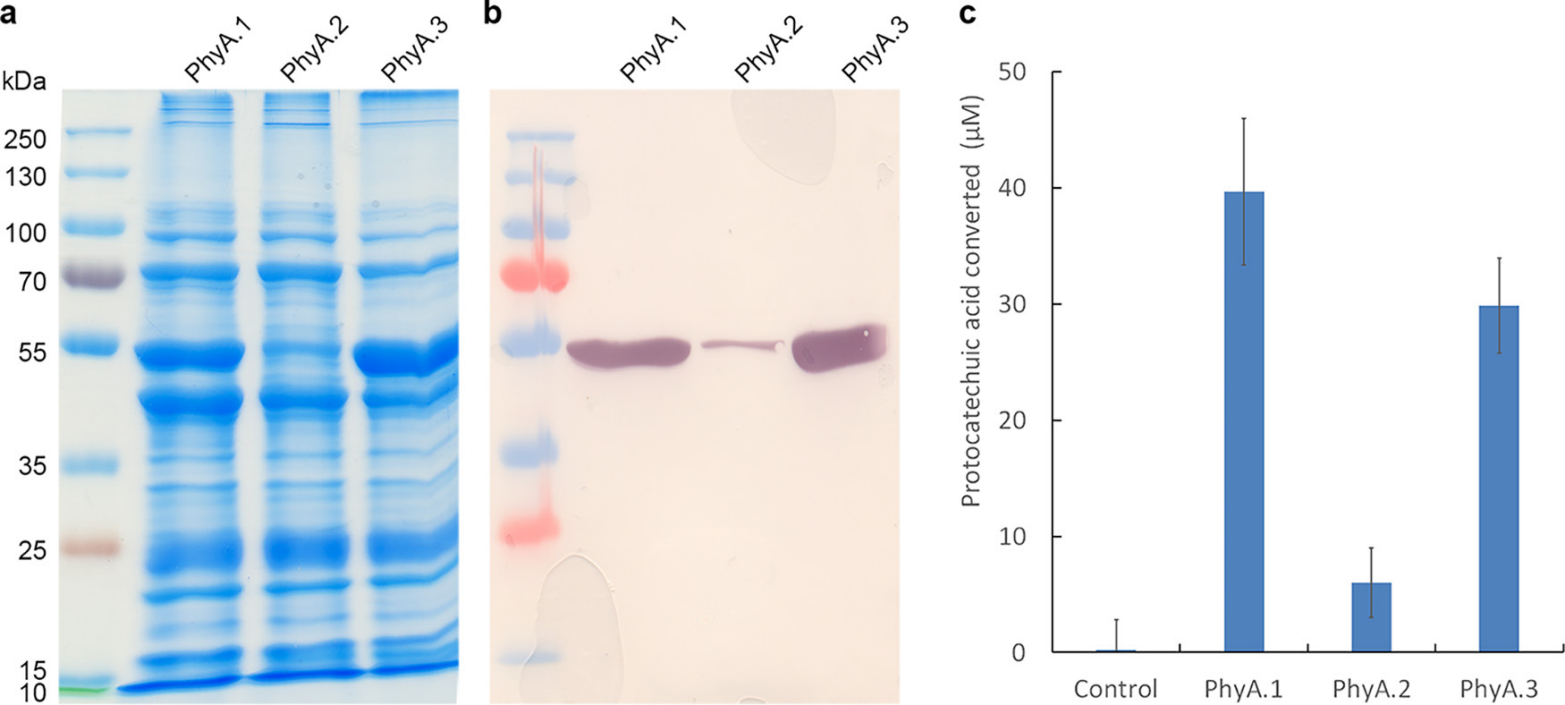
Visualization of PhyA by SDS-PAGE (a), Western blotting (b), and enzymatic activity on protocatechuic acid (c). The enzymatic reaction mixtures contained 100 μM protocatechuic acid and were incubated for 1 h at 30°C.

To investigate the enzymatic activity of PhyA, it was isolated and purified from the three E. coli PhyA-producing strains using immobilized metal affinity chromatography. SDS-PAGE and Western blotting using a monoclonal antibody raised against the histidine tag (Fig. 5a and b) was used to visualize the purified fraction. The estimated molecular mass of PhyA-His, 49.3 kDa, corresponded with the expected mass. The purified PhyA of the three strains was tested for enzymatic activity on protocatechuic acid (Fig. 5c).

### Phylogenetic study of PhyA reveals its conservation in ascomycetes.

**FIG 6:**
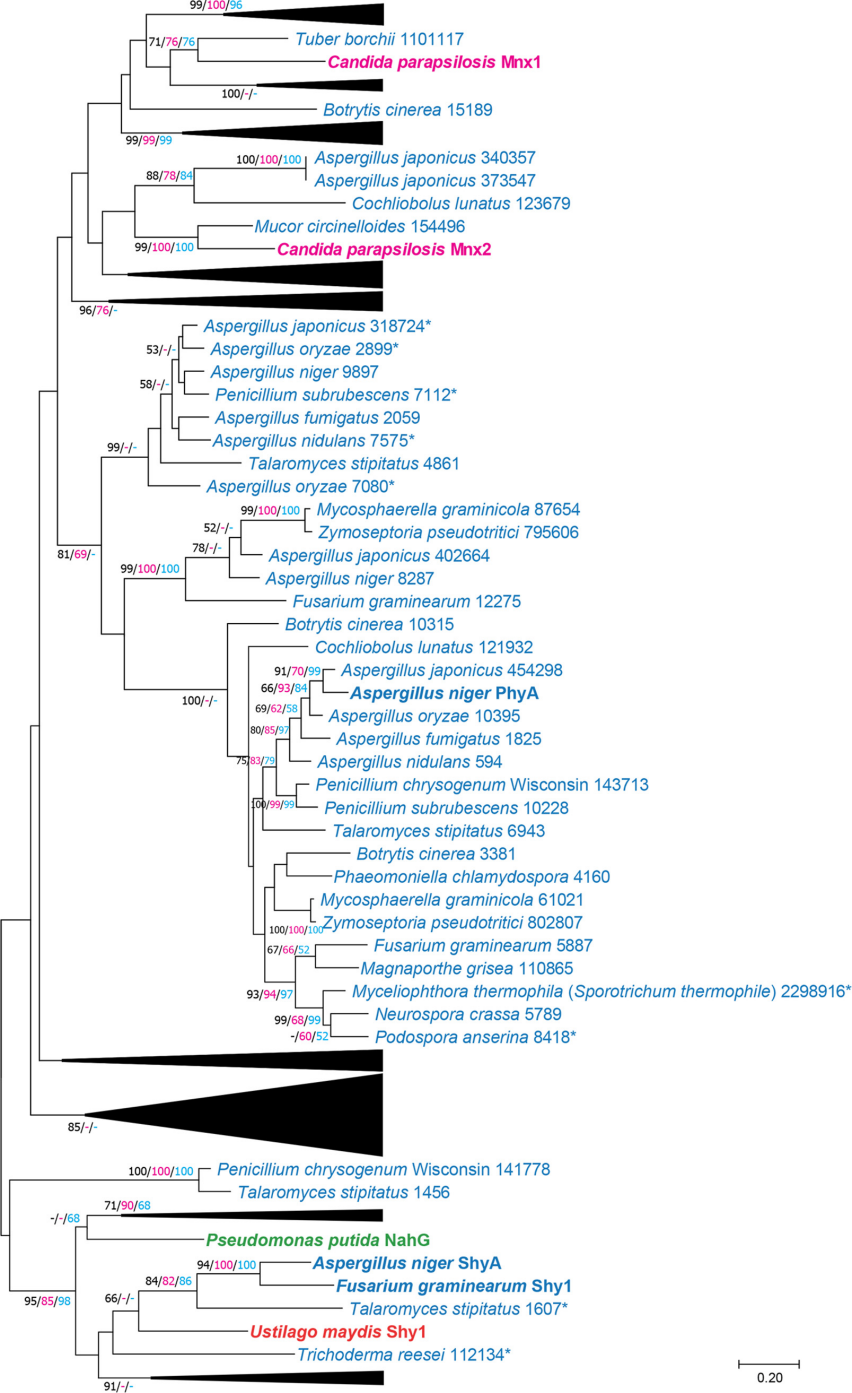
Maximum likelihood (ML; 500 bootstraps) phylogenetic tree of A. niger PhyA homologs from selected fungal genomes. The scale bar shows a distance equivalent to two amino acid substitutions per site. Values over 50% bootstrap support are shown, with ML values in black, neighbor joining values in purple, and minimum evolution values in blue. Characterized enzymes are in bold. Organism names indicated by color: blue, ascomycete fungi; red, basidiomycete fungi; pink, saccharomycetes; green, bacteria. Fungal species names are followed by protein identifiers from JGI (https://mycocosm.jgi.doe.gov/mycocosm/home). Asterisks indicate genes that were curated manually or with the gene prediction software Augustus. The phylogenetic tree without collapsed branches can be found in Fig. S1 in the supplemental material.

To determine if PhyA is conserved in fungi, a phylogenetic study was performed using genes identified in selected ascomycete and basidiomycete genomes using PhyA as the query (Table S2). Most homologs were observed in the Eurotiomycetes, followed by the Dothideomycetes and the Sordariomycetes. No homologs were observed in any of the included yeasts. Characterized salicylate hydroxylases, 4-hydroxybenzoate 1-hydroxylase (Mnx1), and 3-hydroxybenzoate 6-hydroxylase (Mnx2) were added manually to the phylogenetic analysis. PhyA did not cluster with any characterized salicylate hydroxylase nor with Mnx1 or Mnx2 (Fig. 6; Fig. S1), indicating that PhyA represents a novel type of enzyme. Within the clade of PhyA, all included genomes of the Eurotiomycetes (Aspergillus nidulans, Aspergillus oryzae, Aspergillus japonicus, Aspergillus fumigatus, Penicillium chrysogenum, Penicillium subrubescens, and Talaromyces stipitatus) have a homolog of PhyA (Fig. 6; Fig. S1). Other fungi, such as Botrytis cinerea, Phaeomoniella clamydospora, Mycosphaerella graminicola, Zymoseptoria pseudotritici, Fusarium graminearum, Magnaporthe grisea, Myceliophthora thermophila, Neurospora crassa, and Podospora anserina, also had a homolog of PhyA in the clade, but no PhyA homologs were found in *Trichoderma* species nor in any of the basidiomycetes.

10.1128/mBio.00391-21.1 FIG S1 Maximum likelihood (ML; 500 bootstraps) phylogenetic tree of A. niger PhyA homologs from selected fungal genomes. The scale bar shows a distance equivalent to two amino acid substitutions per site. Values over 50% bootstrap support are shown with ML values in black, neighbor joining values in purple, and minimum evolution values in blue. Characterized enzymes are in bold. Organism names indicated by color: blue, ascomycete fungi; red, basidiomycete fungi; pink, saccharomycetes; green, bacteria. Fungal species names are followed by protein identifiers from JGI (https://mycocosm.jgi.doe.gov/mycocosm/home). Asterisks indicate genes that were curated manually or with the gene prediction software AugustusFIG S1, PDF file, 2.9 MB. Copyright © 2021 Lubbers and de Vries. 2021 Lubbers and de Vries. https://creativecommons.org/licenses/by/4.0/ This content is distributed under the terms of the Creative Commons Attribution 4.0 International license.

10.1128/mBio.00391-21.3 TABLE S2 Fungal genomes used for the phylogenetic study of PhyA. Genomes were obtained from JGI MycoCosm (https://mycocosm.jgi.doe.gov/mycocosm/home). Download Table S2, XLSX file, 0.01 MB. Copyright © 2021 Lubbers and de Vries. 2021 Lubbers and de Vries. https://creativecommons.org/licenses/by/4.0/ This content is distributed under the terms of the Creative Commons Attribution 4.0 International license.

## DISCUSSION

Protocatechuic acid is a promising chemical building block for the polymer and pharmaceutical industry (5–8, 12). Here, we showed that the A. niger Δ*phyA* Δ*prcA* double deletion mutant can be used as a fungal cell factory for protocatechuic acid production. Thirteen of the tested aromatic compounds were converted with high efficiency (≥80%) toward protocatechuic acid. Several of these compounds, such as benzaldehyde, *p-*anisic acid, and *p*-anisaldehyde, can be obtained naturally and are available at low cost in large quantities, meaning that these compounds can be used to produce bioprotocatechuic acid. It has been observed that benzoic acid, *p-*coumaric acid, and *p-*hydroxybenzoic acid are released during lignin degradation (32–36). In addition, organic matter found in soil can be an alternative source for *p-*hydroxybenzoic acid, *p-*hydroxybenzaldehyde, and *p*-coumaric acid (37). Agroindustrial by-products and waste streams, such as apple marc, coffee pulp, maize bran, sunflower meal, sugar beet pulp, and wheat straw, contain caffeic acid and/or *p-*coumaric acid, which can be released by ferulic acid esterases from A. niger (38, 39).

Several aromatic compounds can also be found in plant extracts in high concentrations. For example, bitter almond oil from Prunus amygdalus and related species is a rich source of benzaldehyde (28–31, 40) and can be used by A. niger Δ*phyA* Δ*prcA* to produce protocatechuic acid (Table 2). Other sources of benzaldehyde are kernels of almonds, apricots, cherries, peaches, and plums and leaf extracts of Prunus persica (40–42). The aromatic compounds *p*-anisic acid and *p-*anisaldehyde have been extracted from anise, buckwheat, and fennel (43, 44). However, anethole, which is the main aromatic compound in these extracts, was not converted to protocatechuic acid by the Δ*phyA* Δ*prcA* mutant, making these extracts less suitable for protocatechuic acid production.

The conversion of protocatechuic acid to hydroxyquinone has not been described in other ascomycete fungi (24). However, this pathway is possibly present in most aspergilli, since PhyA and HqdA are conserved in this genus (24), although the A. nidulans homologs of PhyA (AN4576) and HqdA (AN5969) were not detected in a previous proteomic study (25). Protocatechuate hydroxylase activity was also observed in the basidiomycete yeast Trichosporon cutaneum (45, 46), but the encoding gene was not identified. Partial characterization of the protocatechuate hydroxylase from *T. cutaneum* revealed that it was highly specific for protocatechuic acid (46). Interestingly, no homologs from basidiomycetes clustered with PhyA, indicating that the protocatechuate hydroxylase from *T. cutaneum* must be encoded by a gene that is not homologous to *phyA*, unless this species obtained a copy of the gene by horizontal transfer from an ascomycete fungus. The *p*-hydroxybenzoate 1-hydroxylase (Mnx1) of Candida parapsilosis was purified and characterized and had activity on several benzoic acid-like substrates, including *p-*hydroxybenzoic acid, protocatechuic acid, and 2,4-dihydroybenzoic acid (47, 48). However, no deletions mutants were made in this species, leaving the *in vivo* metabolic function of this enzyme unknown. Our phylogenetic analysis revealed that PhyA and Mnx1 do not cluster in the same clade, which indicates that Mnx1 may have a different *in vivo* function.

Deleting only *phyA* did not result in a phenotype on protocatechuic acid or related compounds. This demonstrates that the pathway toward hydroxyquinol plays a small role in protocatechuic acid metabolism in A. niger. However, this pathway is essential for A. niger when *prcA* is not present (24). Reduced growth and accumulation of hydroxy-1,4-benzoquinone by the Δ*hqdA* Δ*prcA* mutant on *p*-coumaric acid, caffeic acid, and cinnamic acid revealed that all these compounds are converted to protocatechuic acid, as suggested previously (24). Interestingly, deletion of *phyA* and *prcA* resulted in reduced growth but not in the accumulation of hydroxy-1,4-benzoquinone, indicating that the pathway toward hydroxyquinol is blocked and that no additional protocatechuate hydroxylases are present in A. niger. We previously observed that *p-*anisic acid is converted to *p*-hydroxybenzoic acid, since the deletion of *phhA* resulted in abolished growth on *p-*anisic acid or *p*-anisyl alcohol (24). In Aspergillus japonicus, the conversion of *p*-anisic acid, *p-*anisaldehyde, and *p-*anisyl alcohol was also suggested to lead to *p*-hydroxybenzoic acid (49). In addition, the accumulation of protocatechuic acid by the Δ*phyA* Δ*prcA* mutant now also supports the presence of this metabolic pathway. This indicates that A. niger possesses an unidentified *p-*anisyl alcohol dehydrogenase, *p-*anisaldehyde dehydrogenase, and *p-*anisic acid demethylase.

**FIG 7:**
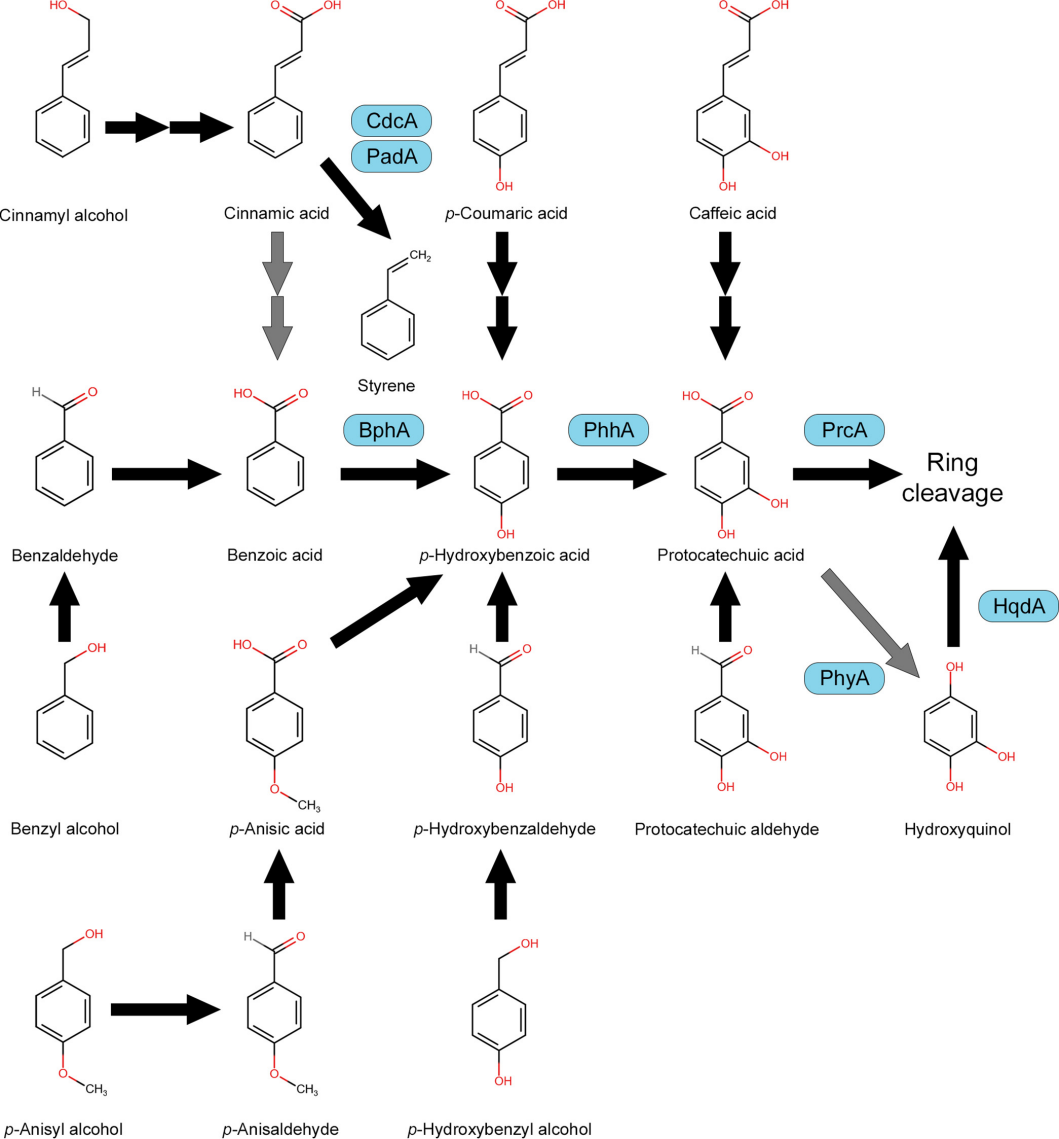
Overview of aromatic compounds converted to protocatechuic acid by A. niger. The identified enzymes are indicated by blue boxes. Gray arrows indicate minor pathways.

Protocatechuic acid accumulated less efficiently when the Δ*phyA* Δ*prcA* strain was grown on cinnamyl alcohol or cinnamic acid. This could be explained by the alternative cinnamic acid pathway of A. niger in which cinnamic acid is decarboxylated by cinnamic acid decarboxylase (CdcA) and coenzyme flavin prenyltransferase (PadA) to styrene (50–53). Deletion of *cdcA* or *padA* results in abolished growth on cinnamic acid (53), while the growth of the Δ*prcA* Δ*hqdA* or Δ*phyA* Δ*prcA* strain was only slightly reduced. In addition, the formation of hydroxy-1,4-benzoquinone was observed when the Δ*prcA* Δ*hqdA* strain was grown on cinnamic acid (Fig. 2), and transcriptome data revealed that cinnamic acid and cinnamyl alcohol induce *bphA*, *phhA*, and *prcA* (53). Therefore, we suggest that cinnamic acid can be converted to protocatechuic acid but that this is a minor pathway, while the pathway to styrene is the major pathway for cinnamic acid conversion in A. niger. Blocking the major pathway might result in the rerouting of cinnamic acid to protocatechuic acid through the minor pathway (Fig. 7).

### Conclusions.

Fungal cell factories are a great alternative approach for the production of aromatic compounds. However, most of the genes of the fungal aromatic metabolic pathways have not been identified, and therefore, strains cannot be readily engineered. By identifying *phyA*, we demonstrated that A. niger can be engineered to produce protocatechuic acid from multiple aromatic compounds related to the H unit of lignin and other related benzoic acids, which provides a reference for many other possible conversions.

## MATERIALS AND METHODS

### Strains, media, and culture conditions.

The A. niger strains used in this study are shown in Table 3. Spores were obtained by growing the fungi on complete medium (CM) (54) plates at 30°C for 4 days and were harvested with 10 ml *N-*(2-acetamido)-2-aminoethanesulfonic acid buffer (pH 6.8). Minimal medium (MM) (55) plates for growth profile experiments were supplemented with aromatic compounds as the sole carbon source, adjusted to pH 6.0, and inoculated with 2-μl droplets containing 10^3^ freshly isolated spores. Due to the toxicity of the aromatic compounds, different concentrations were used for the growth profile. Final concentrations of benzoic acid, cinnamic acid, ferulic acid, and protocatechuic aldehyde were 2 mM each, while final concentrations of caffeic acid, *p*-coumaric acid, *p*-hydroxybenzoic acid, *p*-hydroxybenzaldehyde, protocatechuic acid, and vanillic acid were 5 mM each. All aromatic compounds and chemicals were purchased from Sigma-Aldrich, Saint Louis, MO.

**TABLE 3:** Strains used in this study

Strain	CBS no.	Genotype	Reference
N593 Δ*kusA*	CBS 138852	*cspA1 pyrA* Δ*kusA*::*amdS*	66
Reference	CBS 145984	*cspA1 pyrA*::*pyrG* Δ*kusA*::*amdS*	26
Δ*prcA* mutant	CBS 145165	*cspA1 pyrA* Δ*kusA*::*amdS* Δ*prcA*::*hph*	24
Δ*prcA* Δ*pyrA* mutant	CBS 146349	*cspA1 pyrA*::*pyrG* Δ*kusA*::*amdS* Δ*prcA*::*hph*	This study
Δ*hqdA* mutant	CBS 145839	*cspA1 pyrA* Δ*kusA*::*amdS* Δ*hqdA*::*pyrG*	24
Δ*phyA* mutant	CBS 146351	*cspA1 pyrA* Δ*kusA*::*amdS* Δ*phyA*::*pyrG*	This study
Δ*prcA* Δ*hqdA* mutant	CBS 145840	*cspA1 pyrA* Δ*kusA*::*amdS* Δ*prcA*::*hph* Δ*hqdA*::*pyrG*	24
Δ*phyA* Δ*prcA* mutant	CBS 146352	*cspA1 pyrA* Δ*kusA*::*amdS* Δ*prcA*::*hph* Δ*phyA*::*pyrG*	This study

### Construction of gene deletion cassettes and protoplast-mediated transformation.

Gene deletion cassettes were constructed as described previously (24). The selection marker orotidine 5′-phosphate decarboxylase (*pyrG*) was obtained from Aspergillus oryzae RIB40. A. niger N593 Δ*kusA* and Δ*prcA*::*hph* transformants were obtained through protoplast-mediated transformation and purification as described previously (56). In order to grow all strains under the same condition, the Δ*prcA* Δ*pyrA* mutant was created by replacing the endogenous *pyrA* gene (NRRL3_3466) of the Δ*prcA* strain with the gene deletion cassette containing *pyrG* from A. oryzae. The primers used for the preparation of the deletion cassettes are listed in Table S1 in the supplemental material. Deletion mutants were verified by PCR using the primers listed in Table S1.

10.1128/mBio.00391-21.2 TABLE S1 Primers used in this study. In red are the regions overlapping *pyrG*. Download Table S1, XLSX file, 0.01 MB. Copyright © 2021 Lubbers and de Vries. 2021 Lubbers and de Vries. https://creativecommons.org/licenses/by/4.0/ This content is distributed under the terms of the Creative Commons Attribution 4.0 International license.

### Accumulation test.

Precultures of the A. niger reference strain and the Δ*phyA* Δ*prcA* mutant were performed in 1-liter flasks containing 200 ml MM with 2% d-fructose and inoculated with 2 × 10^8^ freshly harvested spores. The precultures were incubated at 30°C and 250 rpm for 16 h. Mycelium was harvested on Miracloth (Sigma-Aldrich) and washed with MM, after which equal portions of mycelium were transferred to 50-ml flasks containing 10 ml MM supplemented with 2 mM aromatic compound. The cultures were incubated at 30°C and 250 rpm, and supernatant samples were collected after 24 h. These samples were diluted 20 times with acetonitrile and analyzed by HPLC. The reduction of aromatic compounds or formation of the products was monitored by HPLC (Dionex ICS-5000+ chromatography system; Thermo Scientific, Sunnyvale, CA) equipped with an Acclaim mixed-mode WAX-1 LC column (3 by 150 mm; Thermo Scientific) and a UV detector (225 or 280 nm; Thermo Scientific) as described previously (57).

### Production of recombinant PhyA.

A full-length *phyA* gene was synthesized based on the reference sequence (NRRL3_4569) in pET23b containing a C-terminal hexahistidine tag (GenScript Biotech, Leiden, The Netherlands). The pET23b-*phyA* plasmid was used to transform the E. coli protein production strain BL21(DE3) (New England Biolabs, Ipswich, MA). E. coli BL-21 DE3/pET23a-*phyA* was grown overnight in LB medium with 50 μg/ml ampicillin at 37°C and 200 rpm. After incubation, 400 μl of the culture was transferred to a 1-liter flask containing 400 ml LB medium with 50 μg/ml ampicillin and incubated at 37°C and 200 rpm until an optical density at 600 nm (OD_600_) of 0.4 to 0.8 was reached. Then, isopropyl-β-d-thiogalactoside (IPTG) was added at a final concentration of 100 μM, and the culture was further incubated for 24 h at 16°C and 200 rpm. The culture was centrifuged at 3.2 × *g*, 4°C, for 10 min. The supernatant was discarded, and the pellet was dissolved in 20 ml BugBuster protein extraction reagent (Novagen) containing 1 kU lysozyme/ml (Sigma-Aldrich), 25 U benzonase nuclease, and cOmplete EDTA-free protease inhibitor cocktail (Roche, Basel, Switzerland) and incubated for 20 min at 4°C, with shaking. (For lysozyme and benzonase nuclease, 1 unit [U] is the amount of enzyme that will catalyze the transformation of 1 μmol substrate [or product] per minute.) The cell debris was centrifuged at 4°C, and the supernatant containing the soluble fraction of proteins was frozen for further use.

PhyA was isolated for the soluble fraction by purification with a HisTrap FF 1-ml column coupled with the ÄKTA start system (GE Healthcare Life Sciences, Uppsala, Sweden) using the setup described previously (24). After purification, 0.5 mM flavin adenine dinucleotide (FAD) was added to the fractions containing PhyA. The molecular mass of PhyA was calculated *in silico* (https://www.bioinformatics.org/sms/prot_mw.html) and estimated by SDS-PAGE (12% [wt/vol] polyacrylamide gel; Mini-Protean tetra cell, Bio-Rad, Hercules, CA) using the Precision Plus Protein prestained protein standard (Bio-Rad) as a marker. For Western blotting, the proteins were transferred to a nitrocellulose blotting membrane (GE Healthcare, Chicago, IL, USA) and blocked with 5% (wt/vol) skimmed milk in phosphate-buffered saline for 1 h. His-tagged proteins were detected using mouse monoclonal anti-histidine tag antibody (Bio-Rad) conjugated with alkaline phosphatase (Bio-Rad). Proteins were visualized with the BCIP/NBT colorimetric assay (58).

### Enzyme activity assay of PhyA.

Activity assays were performed using cell extracts of E. coli BL21 expressing *phyA*. Cell-free extract of E. coli BL21 harboring an empty vector was used as a negative control. The reaction mixture contained 100 μM McIlvaine buffer, pH 7.0, (59) 100 μM protocatechuic acid, and 40 μl cell extract in a total volume of 1 ml. Reaction mixtures were incubated for 1 h at 30°C and stopped by incubation at 80°C for 10 min. The reaction mixtures were diluted 10 times with acetonitrile and analyzed by HPLC.

An activity assay of purified PhyA toward 100 μM protocatechuic acid was performed in 200-μl reaction mixtures that contained 5 μl purified PhyA, 1 mM NADH, and McIlvaine buffer, pH 7.0 (59). After 1 h of incubation at 30°C, the reactions were stopped by incubation at 80°C for 10 min. A 100-μl reaction mixture was diluted 10 times with acetonitrile and analyzed by HPLC.

### Phylogenetic analysis.

A BLASTP analysis was performed on selected fungal genomes (Table S2) using the amino acid sequence of PhyA as a query. To reduce the amount of insignificant hits, an E value cutoff of e−40 was used, resulting in 164 genes with significant homology to the PhyA gene. The amino acid sequences of ShyA of A. niger (26), ShyI of Fusarium graminearum (60), Shy of Ustilago maydis (61), and 4-hydroxybenzoate 1-hydroxylase (Mnx1) and 3-hydroxybenzoate 6-hydroxylase (Mnx2) of Candida parapsilosis (47, 48) were manually added, and the MUSCLE algorithm was used to create a multiple alignment (62). Several amino acid sequences were curated manually or with the gene prediction software Augustus (Table S3) (63). Maximum likelihood, neighbor-joining, and minimal-evolution trees with 500 bootstrap replications were generated with MEGA X (64).

10.1128/mBio.00391-21.4 TABLE S3 Amino acid sequences used for phylogenetic analysis of products of genes that were curated manually or with the gene prediction software Augustus Table S3, XLSX file, 0.02 MB. Copyright © 2021 Lubbers and de Vries. 2021 Lubbers and de Vries. https://creativecommons.org/licenses/by/4.0/ This content is distributed under the terms of the Creative Commons Attribution 4.0 International license.
