# Supplementary material for: Production of Protocatechuic Acid from p-Hydroxyphenyl (H) Units and Related Aromatic Compounds Using an Aspergillus niger Cell Factory
Source: mBio. 2021 Jun 22;12(3):e00391-21. doi: 10.1128/mBio.00391-21 (PMC8262893; doi:10.1128/mBio.00391-21)
Supplement: FIG S1 [file mbio.00391-21-sf001.pdf]

Supplemental figures

Production of protocatechuic acid from *p*-hydroxyphenyl (H) units and related aromatic compounds using an *Aspergillus niger* cell factory

*Ronnie J.M. Lubbers and Ronald P. de Vries**

Fungal Physiology, Westerdijk Fungal Biodiversity Institute & Fungal Molecular Physiology, Utrecht University, Uppsalalaan 8, Utrecht, 3584CT, The Netherlands.

* Corresponding author, address correspondence to Ronald P. de Vries, r.devries@wi.knaw.nl

Contains 1 figure

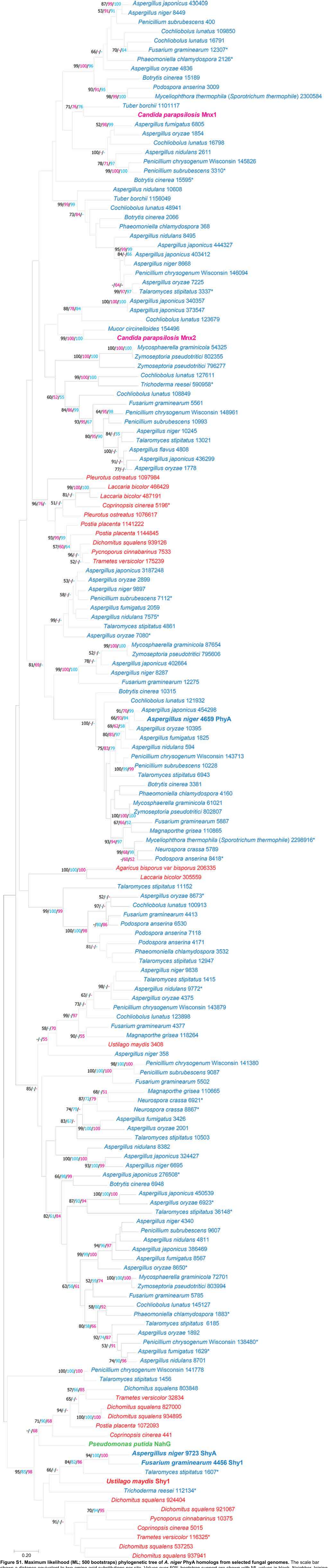

Figure S1. Maximum likelihood (ML; 500 bootstraps) phylogenetic tree of *A. niger* PhyA homologs from selected fungal genomes. The scale bar shows a distance equivalent to two amino acid substitutions per site. Values over 50% bootstrap support are shown with ML values in black, Neighbor Joining values in purple and Minimum Evolution values in blue. Characterized enzymes are in bold. Blue font represents ascomycete fungi, red font basidiomycete fungi, pink font saccharomycetes and green font bacteria. Fungal species names are followed by protein IDs from JGI (https://mycocosm.jgi.doe.gov/mycocosm/home). Genes marked with an asterisk are curated manually or with the gene prediction software, Augustus.
